# Whitecoat Adherence in Patients With Primary Open-Angle Glaucoma

**DOI:** 10.3389/fmed.2022.867884

**Published:** 2022-05-19

**Authors:** Shervonne Poleon, Nouran Sabbagh, Lyne Racette

**Affiliations:** ^1^Department of Optometry and Vision Science, School of Optometry, University of Alabama at Birmingham, Birmingham, AL, United States; ^2^Department of Internal Medicine, University of Alabama at Birmingham, Montgomery, AL, United States; ^3^Department of Ophthalmology and Visual Sciences, School of Medicine, University of Alabama at Birmingham, Birmingham, AL, United States

**Keywords:** glaucoma, medication, whitecoat, adherence, implementation phase

## Abstract

**Purpose:**

Whitecoat adherence refers to improved medication adherence in the days surrounding clinic visits. This may lead to clinical measures that are not representative of those outside of clinical encounters. In glaucoma, whitecoat adherence to prescribed hypotensive therapy may lead to intraocular pressure readings within the target range, which may impact clinical decision-making. We aimed to quantify and identify factors associated with whitecoat adherence.

**Methods:**

In this cohort study, patients with primary open-angle glaucoma were selected from an ongoing longitudinal NIH-funded study if they used hypotensive eyedrops, had a clinic visit during the parent study, and had adherence data during the 28 days evenly bracketing the clinic visit. Adherence within the implementation phase was measured using Medication Event Monitoring System (MEMS) caps. Wilcoxon tests were used to compare mean adherence between the following periods: Pre_14−4_ (days 14 to 4 preceding the clinic visit) and Pre_3−1_ (days 3 to 1 preceding the visit); Post_1−3_ (days 1 to 3 following the clinic visit) and Post_4−14_ (days 4 to 14 following the visit). Analyses were performed in the full sample and in patients with optimal (≥80%, *n* = 49) and suboptimal adherence (<80%, *n* = 17).

**Results:**

Sixty-six patients were included, of which 51.5% were female. Mean age was 70.8 ± 8.1 years. In the 6 months evenly bracketing the clinic visit, mean and median adherence were 86.3% (standard deviation = 17.7) and 95.6% (interquartile range = 21.2), respectively. Overall, mean adherence increased from Pre_14−4_ to Pre_3−1_ (85.5% ± 21.2 to 88.5% ± 23.2, *p* = 0.01) and decreased from Post_1−3_ to Post_4−14_ (87.0 ± 23.9 to 84.9 ± 23.3, *p* = 0.02). In patients with optimal adherence, adherence increased from Pre_14−4_ to Pre_3−1_ (94.0 ± 11.7 to 97.7 ± 7.4, *p* = 0.001) and from Post_1−3_ to Post_4−14_ (95.2 ± 12.0 to 95.4 ± 5.7, *p* = 0.007). Whitecoat adherence was not observed in patients with suboptimal adherence.

**Conclusion:**

We documented the presence of whitecoat adherence in this cohort. Due to its potential impact on clinical outcomes and decisions, providers should remain vigilant for this phenomenon and prioritize it during patient-provider discussions.

## Introduction

Primary open-angle glaucoma (POAG) is a progressive eye disease that is distinguished by connective tissue remodeling at the optic nerve head and characteristic patterns of vision loss. POAG has an estimated global prevalence of over 70 million, and is the leading cause of irreversible blindness worldwide (1). Elevated intraocular pressure (IOP) is the sole modifiable risk factor for glaucoma progression, and daily instillation of hypotensive eyedrops can lower IOP and reduce pressure-induced optic nerve damage. However, despite the effectiveness of ocular hypotensive medications, research indicates that as few as 20% of patients are adherent to prescribed therapy (2). Later investigations by Friedman et al. reported a median adherence rate of 64% based on the analysis of pharmacy claims data for over 13,000 POAG patients (3). These findings are concerning as poor adherence has been associated with faster glaucoma progression (4, 5).

Whitecoat adherence describes patients' tendency to improve their adherence in the days surrounding clinic visits (6). This effect has been documented in several chronic conditions including asthma (7), diabetes (8), and epilepsy (9). In glaucoma, whitecoat adherence is clinically relevant because it can lead to IOP measurements that are unrepresentative of those outside of clinical encounters. IOP readings within the target range may lead clinicians to overestimate treatment effectiveness, which may bias the interpretation of other clinical measures (e.g., visual field imaging results). Furthermore, the obtention of IOP readings within the target range may preclude recommendations for indicated adjunctive therapy or surgical intervention. In this study, we sought to assess whitecoat adherence in patients with POAG and identify factors associated with this phenomenon. We hypothesized that there would be an increase in adherence preceding the clinic visit and a decrease following this visit.

## Materials and Methods

### Study Participants

Ancillary adherence data were obtained from patients enrolled in an NIH-funded longitudinal study (NIH grant EY025756) at the University of Alabama at Birmingham (henceforth referred to as the parent study). Participants in the parent study were required to have a POAG diagnosis, visual acuity better than 20/40, mean deviation better than −12 dB, spherical and cylindrical refraction within 5D and 3D, respectively, and be above age 18 at baseline. Participants with a history of secondary glaucoma, diseases affecting the visual field, intraocular surgery (except uncomplicated cataract or glaucoma surgery), or cognitive impairment were excluded. The parent study was approved by the University of Alabama at Birmingham Institutional Review Board and patients received standard clinical care throughout. All aspects of the study complied with HIPAA regulations and adhered to the tenets of the Declaration of Helsinki.

### Medication Adherence

Medication adherence describes the degree to which actual medication use corresponds with prescribed medication use. The Medication Adherence Reporting Guideline (EMERGE) was developed by the International Society for Medication Adherence (ESPACOMP), and aims to standardize the measurement, analysis, and reporting of medication adherence. EMERGE recognizes three phases of adherence: initiation—when the patient takes the first dose of a prescribed drug, discontinuation—which marks the end of therapy, and implementation—which describes the degree to which patients use their medication as prescribed from treatment initiation to discontinuation (10). In this study, medication adherence was recorded using Medication Event Monitoring System (MEMS) caps (Aardex; Liège, Belgium) during the implementation phase (10, 11). Participants were given one MEMS per prescribed medication and were instructed to store their medication inside the MEMS containers. With this bottle-in-bottle approach, patients were required to open the larger MEMS container to retrieve their medication, replace the medication in the MEMS container after use, and carefully resecure the MEMS caps. Each opening of the MEMS container is logged by the MEMS cap and the electronic measurement serves as a proxy for an instilled eyedrop. Although this method is imperfect, it has been reliably used in previous studies (12, 13). Participants did not receive reminders or feedback on their adherence but were informed at the start of the parent study that the MEMS caps recorded the date and time at which the containers were opened. During research visits, data from the MEMS caps were uploaded into MedAmigo—a web platform for data analysis and visualization—using a MEMS universal serial bus near-field communication reader. Daily adherence was calculated using the following formula:


$$\begin{array}{l} \frac{\text{Number}\;\text{of}\;\text{doses}\;\text{taken}}{\text{Number}\;\text{of}\;\text{doses}\;\text{prescribed}}\;\text{X}\;100\% \end{array}$$

No penalties were applied for taking doses that exceeded the prescribed number, and extra doses were excluded from the calculations. For patients with multiple medications, daily adherence was calculated per medication and averaged across the total number of medications. Adherence data were downloaded from MedAmigo and reviewed to ensure that all changes in regimen during the parent study were accounted for. Adherence data for the first 2 months of the parent study were excluded from this analysis to minimize the influence of the Hawthorne effect, which is more marked at the start of the monitoring period (9).

### Clinic Visits

To be included in this analysis, participants from the parent study needed to have attended at least one clinic visit with their eye care provider between May 10, 2018 (the start of the parent study) and March 13, 2020 (date of the declaration of the Covid-19 pandemic). We excluded clinic visits after March 13, 2020, as research indicates that adherence in glaucoma was negatively affected during the COVID-19 pandemic (14, 15). We reviewed participants' clinical charts to identify eligible clinic visit dates. To limit the influence of whitecoat adherence associated with research visits during the parent study, we excluded clinic visits that occurred within 14 days of a research visit (16). For each patient, we selected the first eligible clinic visit date and calculated mean daily adherence for each of the days in the 28-day period evenly bracketing this date.

### Factors Associated With Whitecoat Adherence

To identify factors associated with whitecoat adherence, we included the demographic, clinical, and psychological data collected during the parent study. Demographic factors included patients' self-reported age, race, gender, education level, marital status, employment level, and income level. Clinical factors included mean adherence, number of prescribed ocular medications, and regimen complexity. We operationalized regimen complexity as the number of daily eyedrop instillations multiplied by the number of prescribed ocular medications (17). Mean adherence was computed for each participant over the 180-day period evenly bracketing the date of the clinic visit. Psychological factors included patients' perceptions of glaucoma, which were assessed using the Brief Illness Perception Questionnaire (BIPQ) (18). The BIPQ uses a 0–10 Likert-type scale to assess eight domains related to illness perception: consequences, timeline, personal control, treatment control, identity, concern, emotional representation, and coherence. Subscale and total BIPQ scores were computed.

### Statistical Analysis

**Figure 1:**
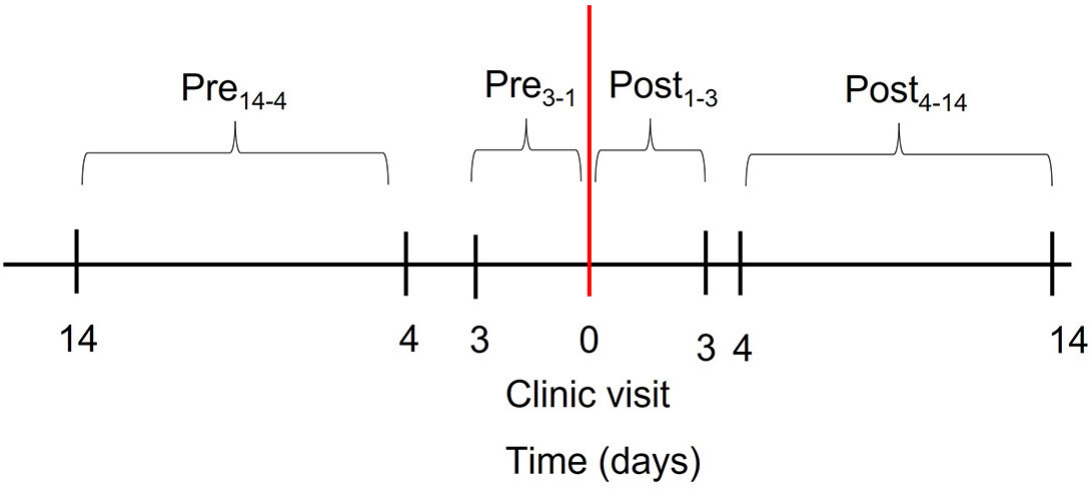
Study design. Study periods within the 28 days evenly bracketing the clinic visit (red line) are depicted by black brackets.

Based on previous work showing an increase in adherence within the 3-day period preceding the clinic visit (9, 19), we used Wilcoxon signed-rank test to identify significant differences in mean adherence between the following study periods: Pre_14−4_ (days 14 to 4 preceding the clinic visit) and Pre_3−1_ (days 3 to 1 preceding the clinic visit); Post_1−3_ (days 1 to 3 following the clinic visit) and Post_4−14_ (days 4 to 14 following the clinic visit). Figure 1 depicts the study design and study periods. As research indicates that whitecoat adherence may vary by adherence level (20), we stratified participants using an 80% threshold (21), where adherence <80% was deemed to be suboptimal and adherence ≥80% was deemed to be optimal. Wilcoxon signed-rank test was repeated in each adherence group. Lastly, we performed univariate linear regression to identify factors associated with whitecoat adherence, which we operationalized as an increase in adherence from Pre_14−4_ to Pre_3−1_ or a decrease in adherence from Post_1−3_ to Post_4−14._ Analyses were performed in JMP Pro (version 16), and alpha was set at 0.05.

## Results

A total of 66 participants were included in this analysis. Table 1 presents participant characteristics. Mean age was 70.8 ± 8.1 years and mean number of prescribed hypotensive medications was 1.6 ± 0.7. Approximately 51.5% of participants were female and 57.6% self-reported as White. Fifty-nine percent of participants attained a baccalaureate degree or higher, and approximately 30% reported a household income of $60,000 or more. Mean adherence was 86.3% ± 17.7 compared to the median value of 95.6% (interquartile range, IQR = 21.2) Median BIPQ total score was 27 (IQR = 12). The maximum possible BIPQ total score was 80, with higher BIPQ total scores indicating a more daunting outlook on glaucoma.

**Table 1:** Participants clinical, demographic, and psychological characteristics.

**Study variable**	
Age (years) mean ± SD	70.8 ± 8.1
Number of ocular medications, mean ± SD	1.6 ± 0.7
Medication adherence, mean ± SD (median, IQR)	Percentage (%)
Overall	86.3 ± 17.7 (95.6, 21.2)
Optimal (*N* = 49)	95.1 ± 5.5 (97.8, 6.8)
Suboptimal (*N* = 17)	60.7 ± 15.9 (64.6, 18.2)
Gender	Percentage (%)
Female	51.5
Male	48.5
Race	Percentage (%)
White	57.6
Black	42.4
Education level	Percentage (%)
High school/Some college	40.9
Baccalaureate	31.8
Graduate	27.3
Marital status	Percentage (%)
Married	65.2
Not married	34.8
Employment level	Percentage (%)
Employed full-time	28.8
Not employed full-time	71.2
Household income	Percentage (%)
$60,000 or less	42.5
Above $60,000	30.3
Declined to answer or unknown	27.2
BIPQ subscale scores	Median (IQR)
Total BIPQ score	27 (12)
Consequences	1 (2.3)
Timeline	10 (0.25)
Personal control	2 (4.3)
Treatment control	1 (2)
Identity	0.5 (3.3)
Concern	8 (5)
Emotional representation	1 (2)
Coherence	1 (2.3)

**Figure 2:**
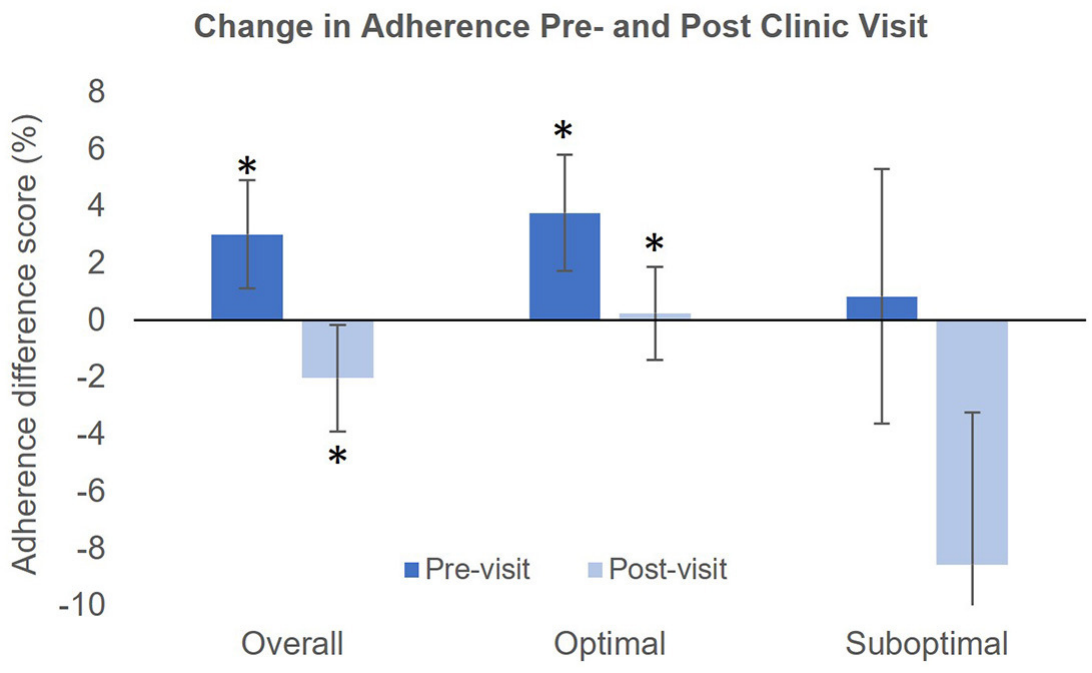
Magnitude of the change in adherence pre- and post-clinic visit in the study population. Asterisks indicate significant differences between the Pre_14−4_ and Pre_3−1_ or between Post_1−3_ and Post_4−14_.

Table 2 presents mean adherence during each study period in the overall sample, as well as in patients with optimal and suboptimal adherence. As depicted in Figure 2, patients with optimal adherence showed an increase in adherence both prior to and after the clinic visit (Pre_14−4_ to Pre_3−1_: 94.0 ± 11.7 to 97.7 ± 7.4, *p* = 0.001; Post_1−3_ to Post_4−14_: 95.2 ± 12.0 to 95.4 ± 5.7, *p* = 0.007). There was no significant change from Pre_14−4_ to Pre_3−1_ (*p* = 0.69) or from Post_1−3_ to Post_4−14_ (*p* = 0.32) in patients with suboptimal adherence. In the entire sample, mean adherence increased from Pre_14−4_ to Pre_3−1_ (85.5% ± 21.2 to 88.5% ± 23.2, *p* = 0.01) and decreased from Post_1−3_ to Post_4−14_ (87.0 ± 23.9 to 84.9 ± 23.3, *p* = 0.02). Overall, there was a small but significant increase of 3.0% ± 15.2 (range = −36.4 to 63.64%) prior to the clinic visit and a decrease of 2.0% ± 15.0 (range = −36.4 to 54.5%) afterwards. Among only patients with whitecoat adherence prior to the clinic visit (*n* = 29), there was an increase of 13.4% ± 14.0 (range = 1.5 to 64%). Among patients with whitecoat adherence after the clinic visit (*n* = 27), there was a decrease of 13.4% ± 13.0 (range = −0.7 to −54%).

**Table 2:** Mean adherence per study time point.

	**Pre_**14−4**_**	**Pre_**3−1**_**	**P value**	**Post_**1−3**_**	**Post_**4−14**_**	***P*-value**
Overall	85.5 ± 21.2	88.5 ± 23.2	0.01	87.0 ± 23.9	84.9 ± 23.3	0.02
Optimal	94.0 ± 11.7	97.7 ± 7.4	0.001	95.2 ± 12.0	95.4 ± 5.7	0.007
Suboptimal	60.9 ± 23.7	61.8 ± 31.7	0.69	63.2 ± 33.1	54.7 ± 28.0	0.32

In the full sample, no clinical or demographic variables were associated with whitecoat adherence before or after the clinic visit. This was also true in patients with suboptimal adherence. In patients with optimal adherence, lower education level was associated with whitecoat adherence after the clinic visit (B = −4.0, *p* = 0.046). Table 3 presents the associations between whitecoat adherence and BIPQ scores. In the full sample, a significant negative association was observed between whitecoat adherence before the clinic visit and BIPQ total score (B = −0.40, *p* = 0.01). This was also true in the optimal adherence group (B = −0.39, *p* = 0.03). The personal control and treatment control subscales of the BIPQ were negatively associated with whitecoat adherence prior to the clinic visit in the full sample (B = −1.41, *p* = 0.02 and B = −1.96, *p* = 0.048, respectively). Similar associations were observed in patients with optimal adherence (Personal control: B = −1.52, *p* = 0.02, Treatment control: B = −2.06, *p* = 0.04).

**Table 3:** Coefficients for the relationship between BIPQ scores and whitecoat adherence.

**BIPQ Scores**	**Pre_14−4_ to Pre_3−1_ estimate**			**Post_1−3_ to Post_4−14_ estimate**		
	**Overall**	**Optimal**	**Suboptimal**	**Overall**	**Optimal**	**Suboptimal**
Total BIPQ Score Consequences Timeline Personal control Treatment control Identity Concern Emotion representation Coherence	–**0.40** −1.13 −0.21 –**1.41** –**1.96** −1.08 −0.47 −1.16 −0.64	–**0.39** −1.00 −0.04 –**1.52** –**2.06** −1.16 −0.47 −1.46 −0.56	−0.48 −1.29 −0.65 −1.12 −1.48 −0.80 −0.28 −0.49 −0.73	0.11 −0.54 0.97 0.76 −0.68 0.05 0.38 0.57 −0.08	**0.29** 1.24 0.94 0.33 0.18 1.13 **1.26** −0.53 0.71	−0.81 −**3.89** 1.34 1.42 −4.97 −3.56 −**5.46** 3.26 −2.52

*Bolded values represent statistically significant estimates*.

In the period following the clinic visit, no significant associations were observed between BIPQ subscale scores and whitecoat adherence in the full sample. Among patients with optimal adherence, there was a positive association between BIPQ total score and whitecoat adherence (B = 0.29, *p* = 0.04), as well as between the concern subscale score and whitecoat adherence (B = 1.26, *p* = 0.01). Among patients with suboptimal adherence, there was a negative association between the concern subscale score and whitecoat adherence (B = −5.46, p = 0.01), as well as between the consequences subscale score and whitecoat adherence (B = −3.89, *p* = 0.05).

## Discussion

Whitecoat adherence has previously been documented in several chronic conditions (7, 8, 22). In this study, we reported the presence of whitecoat adherence in patients with POAG, which supported our hypothesis. We documented higher adherence within 3 days of the clinic visit, consistent with findings by Modi et al. (9) who reported a significant increase in the use of anti-epileptic drugs in the 3 days preceding the clinic visit. A similar finding was documented by Zueger et al. (19) who found that a significantly higher number of insulin boluses were administered in the 3 days prior to clinic visits. In glaucoma, Kass et al. also observed a significant increase in adherence, specifically within 24 h of the clinic visit (23).

We observed whitecoat adherence within 3 days of the clinic visit in the overall sample as well as in patients with adherence ≥80%. This suggests that patients with higher adherence may also have higher levels of healthcare engagement, which would prompt them to place greater emphasis on their adherence, particularly prior to the clinic visit. However, Okeke et al. reported a whitecoat effect in patients with adherence below 75% (20). This discrepancy could be due to differences in the characteristics of the two cohorts. Patients included in our analysis were also participants in a 2.5-year longitudinal study, and may have higher levels of healthcare engagement compared to patients not engaged in clinical research. In this study, whitecoat adherence was not observed in patients with suboptimal adherence. This could potentially be due to the small size of this group. Although there was a large decrease in adherence after the clinic visit, high variability in adherence measurements in this group reduced our ability to detect a significant effect. Overall, there was a mean increase of 3% prior to the clinic visit and a mean decrease of 2% afterwards. Among only patients who demonstrated a whitecoat effect, there was a mean change of ±13.4% before and after the clinic visit. The magnitude of this change is sufficiently large to be of concern clinically.

Medication adherence is a complex and dynamic behavior as up to five distinct patterns have been observed in POAG (24–27). During a given period for instance, highly adherent patients may take drug holidays. As such, metrics such as mean and median adherence may not adequately capture gaps in medication use, resulting in undetected periods of uncontrolled IOP. Hypotensive eyedrops lower IOP per 12 or 24-h, which can mask periods of uncontrolled IOP prior to the clinic visit. In the absence of regular visual field testing—which may not be requested if IOP appears to be controlled—glaucomatous vision loss may not be easily detected. This line of thought is consistent with reports of progressive worsening of the visual field with IOP levels seemingly at or below the target when measured in the clinic (20). As suboptimal adherence has also been associated with faster rates of vision loss (5), it also has a significant negative impact on clinical and patient-reported outcomes. Poor and non-adherence may go undetected by providers, and the opportunity to prescribe alternative therapies or deliver interventions that could improve adherence and delay further worsening of the visual field may also be missed.

Whitecoat adherence can be attributed to several factors. For instance, increasing proximity to the clinic visit likely serves as a reminder for patients to instill their medication and prevent disease progression. Additionally, the impending clinic visit signals imminent face-to-face contact with the eye care provider. This may motivate patients to increase their adherence in an effort to avoid providers' disapproval (20). In the clinic, medication adherence is often assessed via patient reports and is frequently overestimated (28). Whitecoat adherence may contribute to this effect as patients' assessments may be biased in favor of more recent adherence behavior. As research indicates that the whitecoat effect may be more marked at the beginning of treatment (9), newly diagnosed patients should monitored more closely for poor adherence and more objective methods should be employed where possible. In addition to increased monitoring, poor adherence may be addressed in the clinic by improving the patient-provider relationship. Research conducted in a cohort of hypertensive patients suggests that patients who engaged in active vs. passive decision-making had higher adherence (29). Providers may employ a shared decision-making approach that encourages patients to become more involved in their care. This may strengthen and lengthen the patient-provider relationship, which has also been associated with higher adherence (29). The patient-provider relationship has been identified as a facilitator of good adherence (30), and research has shown that non-adherent patients were less likely to believe that their eye doctors spent sufficient time talking with them about their eye condition (31). Increased focus on patient education regarding the clinical impact of poor adherence may also help to increase engagement in eye care and improve adherence to prescribed medical therapy.

In this analysis, we found that lower BIPQ total score was associated with whitecoat adherence prior to clinic visits. Patients with a less daunting view of glaucoma (lower BIPQ total score) may experience lower levels of psychological stress, which may lead to higher levels of engagement in eye care and ultimately higher adherence. This is consistent with the finding of Jiang et al. (32) who reported that BIPQ total score was inversely associated with medication adherence The personal control and treatment control subscales of the BIPQ describe patients' perceived level of control over their illness and degree to which treatment can help their illness, respectively. These scales are inverted, with lower scores representing higher perceived ability. Lower scores were associated with whitecoat adherence prior to the clinic visit. This finding may be explained by patients' higher levels of confidence in their control of the disease and the effectiveness of treatment, which may motivate them to improve their adherence as the clinic visit approaches. Lower scores on both subscales are analogous to higher self-efficacy and treatment efficacy, which have been linked with higher adherence (33, 34).

The illness concern subscale measures patients' level of concern about their condition. Lower scores were associated with lower adherence after the clinic visit in the optimal adherence group compared to higher adherence after the visit in the suboptimal adherence group. Thus, the clinic visit may have a different impact on these patient groups. Patients with optimal adherence and low levels of concern about glaucoma may feel secure in their management of the condition and may not be driven to improve their adherence after the clinic visit. However, for patients with suboptimal adherence, the clinic visit may reinforce the need to control IOP and prevent vision loss, leading to higher adherence after the visit. This could also explain the positive association between illness consequences score—which describes the perceived impact of illness on one's life—and whitecoat adherence after the clinic visit. Given the ramifications of whitecoat adherence on clinical outcomes, prioritizing this topic during patient-provider discussions is critical for helping patients to maintain high levels of adherence throughout the course of treatment.

This study has several strengths. We assessed adherence using electronic monitoring, which provides objective measurements. A drawback of this approach is that patients using electronic monitors are susceptible to the Hawthorne effect, which can produce artificially high measurements. However, we guarded against this by excluding the first 2 months of monitoring data. We were also able to identify psychological factors associated with whitecoat adherence, providing potential insight into this phenomenon. This study is not without limitations, which include the surrogate nature of the adherence data collected with MEMS. However, this method has been shown to yield more accurate data than self-report. While direct observation would be more accurate, it is not practical in glaucoma where patients instill eyedrops daily. A second limitation associated with the longitudinal cohort used in this study was our inability to assess whitecoat adherence over multiple clinic visits. This was not possible as the number of research visits during the parent study reduced the number of eligible clinic visits for our analysis. Another limitation is the possible presence of a whitecoat effect throughout the parent study. Participants enrolled in the parent study were required to complete 12 research visits over a 2.5-year period. This may have contributed to a consistent whitecoat effect throughout the parent study which could have minimized the magnitude of the effect detected during the period of our analysis. We minimized this limitation by ensuring that no research visits occurred within the 28-day period evenly bracketing the clinic visit. Nonetheless, our ability to detect whitecoat adherence in this cohort suggests that the effect may be even more marked in the wider patient population. Lastly, the relatively small number of patients with suboptimal adherence reduced our ability to detect a whitecoat effect in this group.

In this study, we documented a significant increase in adherence within 3 days of the clinic visit. This supported our hypothesis. Beliefs about personal control, treatment control, illness concern, and illness consequences were associated with whitecoat adherence. Providers should remain vigilant for these factors and prioritize discussions regarding medication adherence during clinic visits. Future research should assess whitecoat adherence using electronic monitoring to determine whether this finding is consistent in the wider patient population.

## Data Availability Statement

The raw data supporting the conclusions of this article will be made available by the authors, without undue reservation.

## Ethics Statement

The studies involving human participants were reviewed and approved by University of Alabama at Birmingham Institutional Review Board. The patients/participants provided their written informed consent to participate in this study.

## Author Contributions

SP: study design, data analysis, and manuscript preparation. NS: data preparation, and manuscript preparation. LR: study design, data analysis, manuscript preparation, and supervision of all aspects of research. All authors contributed to the article and approved the submitted version.

## Funding

This study was supported by the National Eye Institute of the National Institutes of Health (Bethesda) under award numbers R01EY025756 and P30EY003039. The study was also supported by an unrestricted grant from Research to Prevent Blindness. The sponsors had no role in the design or conduct of this research.

## Conflict of Interest

LR is a scientific advisor for Olleyes, Inc. The remaining authors declare that the research was conducted in the absence of any commercial or financial relationships that could be construed as a potential conflict of interest.

## Publisher's Note

All claims expressed in this article are solely those of the authors and do not necessarily represent those of their affiliated organizations, or those of the publisher, the editors and the reviewers. Any product that may be evaluated in this article, or claim that may be made by its manufacturer, is not guaranteed or endorsed by the publisher.
